# The Bell Pattern: A Novel Breast Incision Approach to Skin-Reducing Mastectomies

**DOI:** 10.1093/asjof/ojz031

**Published:** 2019-10-23

**Authors:** William B Albright, Patrick J Hawkes

## Abstract

**Background:**

As immediate direct to permanent implant-based breast reconstruction (IBBR) continues to gain in popularity, surgeons seek to apply these techniques to patients with large or ptotic breasts. A new bell pattern skin excision is described and limits major complications in this high-risk population.

**Objective:**

The authors describe a novel skin excision pattern for patients with large or ptotic breasts who desire IBBR and assess its safety. The authors also evaluated the ability of the pattern to account for intraoperative developments.

**Methods:**

This retrospective analysis of a single surgeon’s experience included 17 consecutive patients (31 breasts) with large or ptotic breasts undergoing skin-reducing mastectomy with attempted utilization of the bell pattern approach and IBBR with acellular dermal matrix.

**Results:**

Mean age was 50 years, mean body mass index was 27.4 kg/m^2^, and mean breast specimen weight was 683 g. A bell pattern excision was planned for all breasts preoperatively. Three breasts (10%) required an alternative closure pattern due to intraoperative ischemia (*n* = 1), or additional oncologic resection (*n* = 2). The pattern successfully accommodated flap ischemia in 8 (26%) other breasts. After a median follow-up of 5.1 months, the number of bell pattern breasts with major and minor complications was 0 (0%) and 9 (32%), respectively. The most common minor complication was seroma (*n* = 5, 18%), and minor incision wound (*n* = 3, 11%). There were no reconstruction failures utilizing the bell pattern.

**Conclusion:**

The bell pattern approach is a safe and adaptable alternative to traditional skin-reducing mastectomy in patients with large or ptotic breasts.

**Level of Evidence: 4:**

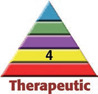

Immediate breast reconstruction continues to grow in utilization from an estimated 27% of mastectomy patients in 2005 to 43% by 2014.^1^ Approximately 101,657 reconstructions were performed by ASPS members in the United States in 2018.^2^ In 2002, implant-based breast reconstruction (IBBR) surpassed autologous reconstruction and accounted for a majority of all reconstructions in the United States,^3^ a gap that continues to widen.

As technology and techniques have improved, surgeons have expanded the surgical indications for IBBR to patients traditionally considered too risky for prostheses,^4^ namely those with a high body mass index (BMI) and large and/or ptotic breasts. However, this expansion coincides with another important trend: outcomes and cost under ever increasing scrutiny. For many surgeons, the perceived risks and cost of complications has now led them to discourage large breasted patients from undergoing immediate IBBR. The costs are real: In 2014, the estimated institutional cost alone of a failed IBBR in the United States was $32,500.^5^ The risks are well founded, as surgeons report difficulty managing the redundant mastectomy skin with less reliable blood supply^6,7^ as seen with current skin-reducing mastectomy (SRM) techniques.

In patients undergoing SRM, elliptical excision has been criticized for poor aesthetic outcomes, such as flattening projection, and leaving prominent medial and lateral “dogears” ^8^ made worse in very large or ptotic breasts. To address these shortcomings, surgeons have reported numerous alternative excision patterns, including oblique,^9^ circumvertical,^10,11^ trans-vertical,^12^ Lazy S,^13^ double-mirrored omega,^14^ and inframammary skin sparing patterns.^15^ Yet others instead discourage a single-stage approach and recommend performing staged procedures only (ie, oncoplastic reduction prior to mastectomy). Not only does this necessitate multiple surgeries each with inherent risks and higher overall healthcare costs, it may delay onset of oncologic treatments^16^ in high-risk groups.

Perhaps the most widely used SRM pattern in immediate IBBR is the Wise pattern with or without a caudally based dermal flap.^17–25^ When compared to an ellipse, the Wise pattern produces a more projecting breast shape and allows management of horizontal skin excess.^23^ However, as is widely reported,^6,21,22,26^ this technique is particularly susceptible to flap ischemia, skin necrosis, and implant failure (Figure 1). Although not often discussed, the pattern is difficult to adjust to preoperative breast scars and intraoperative flap ischemia. When utilizing a dermal flap, extensive de-epithelialization requires additional operative time and increases risk for epidermal inclusion cysts.^23^

**Figure 1.**
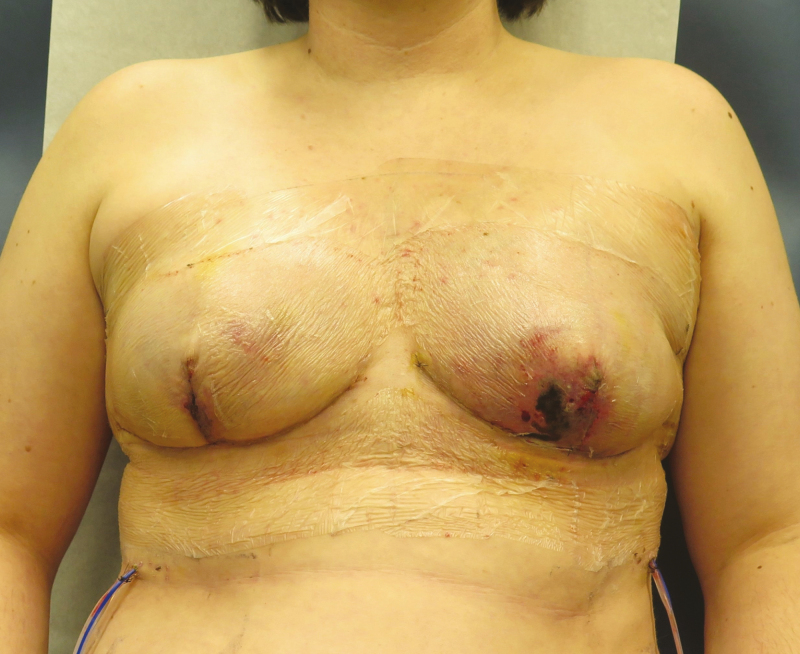
A 40-year-old woman 10 days after bilateral Wise pattern skin reducing mastectomies with evolving skin necrosis bilaterally, who subsequently developed infection bilaterally necessitating debridement, explantation, and delayed reconstruction.

The purpose of this study is to describe a novel skin excision pattern for SRM in women seeking immediate IBBR devised: The bell pattern. We report a single surgeon’s outcomes utilizing this technique with specific reference to preoperative and intraoperative adjustability of the skin excision pattern, and frequency of postoperative complications. Finally, the complication rates and aesthetic outcomes of both the bell pattern and the Wise pattern will be compared.

## METHODS

A retrospective review of all patients undergoing bell pattern SRM with immediate IBBR by a single surgeon between May 2018 and May 2019 was performed. Patient charts were reviewed to obtain necessary data including demographics, surgical, and postoperative outcomes. The study was reviewed and approved by University of Iowa Hospitals and Clinics IRB committee.

Patients were considered for bell pattern excision if they presented with Regnault Grade 0-3 ptosis, were undergoing non-nipple-sparing SRM and desired prepectoral IBBR. A bell pattern was not offered if the patient had (a) grade 0 ptosis with small breast volume more amenable to a traditional elliptical excision, or (b) desired nipple-sparing mastectomy, or (c) had any of the following risk factors: BMI >35 kg/m^2^, active nicotine use, or previous breast radiation. These preoperative risk factors were selected by the primary author as exclusion criteria for IBBR with SRM. Patients with history of nicotine use within the past 2 years were screened with blood cotinine level prior to surgery. Of the 2 patients with recent history of nicotine use and negative nicotine tests, both resumed nicotine use during the immediate postoperative period. No other exclusion criteria were utilized.

**Figure 2.**
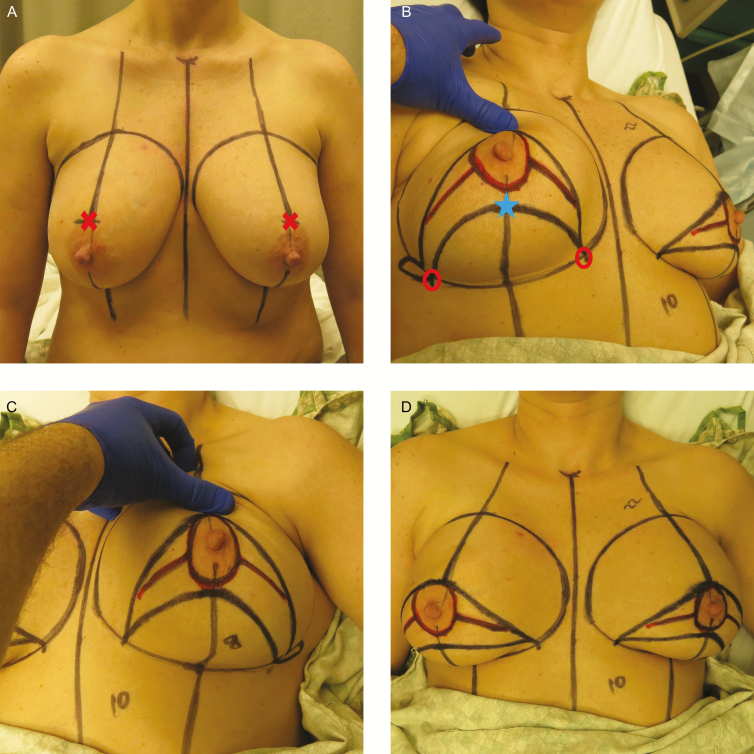
An example of preoperative markings. (A) This 45-year-old female patient was marked in the seated position with midline and breast meridian marked. Apex of superior arch is marked with an “X.” (B) The patient marked in supine position, showing caudal right breast markings. Flap base width is shown between the “circle” markings. Apex of inferior arch is marked with a “star.” (C) The patient showing caudal left breast markings. (D) Final markings with patient in supine position.

Patients are initially marked in the seated position. The nipple-areola-complex (NAC) is encircled for the mastectomy resection access incision. The breast meridian is drawn and adjusted as needed to align with the apex of the areola. For bilateral cases, the more cephalad NAC is identified, measured from the sternal notch, and marked along the meridian (Figure 2A, marked with an “x”). This mark is transposed to the contralateral breast and adjusted to ensure marks are level in the horizontal plane. This mark identifies the top of the superior incision arch.

The rest of the markings are best made while the patient is supine with the breast centered over its footprint. The caudally based inferior mastectomy flap (lower arch) will be created next. First the flap base width (FBW) must be calculated based on the patient’s breast base width (BBW) according to the formula (BBW - 2 cm = FBW). The FBW is centered on the breast meridian at the inframammary fold and marked along the IMF (Figure 2B, marked with a circle). Next the lower pole of the breast (IMF: areola) is measured at the meridian bilaterally. The more caudal NAC (the shorter IMF:areola) is identified, and marked. This point will mark the top of the inferior incision arch (Figure 2B, marked with a star). The lower incision line can then be marked starting at this caudal meridian point and curving medially and laterally to the FBW marks along the IMF.

Finally, the superior incision line is marked starting at the cephalad meridian mark and curving medially and laterally down to the inferior FBW marks to join the inferior arch; thus, creating the upper arch of the bell pattern. There will now be 2 symmetric arches drawn on the breasts with the NAC sandwiched between the lines and both ending at a point on along the IMF (Figure 2B-D). An additional lateral extension can be marked to help eliminate the lateral dogear if necessary. If additional access is needed for the mastectomy, short incisions medial and lateral to the NAC can be made, as long as these incisions stay between the upper and lower bell lines.

After mastectomy completion, flap perfusion is assessed in all patients using indocyanine green laser angiography (ICGLA) (SPY or PDE). Attempts are made to adjust the proposed bell pattern excision to incorporate underperfused areas. If adequate perfusion, a sterile implant sizer is placed into the pocket and the proposed upper and lower incision marks are loosely approximated to ensure minimal skin tension.

In this study, a complete acellular dermal matrix (ADM) (Alloderm) wrap for tissue-expander breast reconstruction (TEBR), and an anterior ADM wrap for direct to implant (DTI) reconstruction were used.

Once the prosthesis is secured, the proposed bell incisions are again loosely pinched to ensure adequate soft tissue prior to committing to bell incisions. After the bell pattern skin is excised, any lateral skin redundancy can then be estimated by tailor tacking and excised in a posterior direction.

Prior to closure, 10cc 0.25% bupivacaine plain is flushed topically, followed by placement of two 15Fr round Blake drains into each breast pocket (one directed medially and the other directed laterally). At this time, *assuming confidence with initial flap perfusion*, internal pocket-defining tacking sutures can be placed. Typical locations for internal tacking sutures include (1) resetting IMF when undermined during resection, and (2), in obese patients with heavy full axilla, advancing posterior tissue anteriorly and anchoring to serratus fascia to offload anterior skin closure tension.

The incisions are closed in layers using simple interrupted 3-0 Vicryl deep dermal sutures starting at the meridian. The medial and lateral closures proceed by dividing distances to the IMF in half and continuing so as to minimize pleating. Finally, a 4-0 Monocryl subcuticular stitch is run. Prior to application of a skin adhesive, the fluorescent angiography is again performed in all patients to ensure adequate perfusion of the remaining skin, and any necessary revision performed on-the-table. Immobilization of the breast construct with application of Tegaderm dressing applied directly to the anterior chest is performed in all patients.

## RESULTS

Between May 2018 and May 2019, 31 breast reconstructions in 17 patients were performed by a single surgeon (W.B.A.) with planned bell pattern non-nipple-sparing SRM (Table 1). Patients had an average follow-up of 5.1 months (range, 1-13 months). Fourteen patients underwent bilateral reconstructions, and 3 patients underwent unilateral reconstruction. All breasts received a prosthetic-based reconstruction and all prostheses were placed in a prepectoral pocket with human origin ADM. The average patient was 50 years old (range, 33-68 years), had a BMI of 27.4 kg/m^2^ (range, 20.4-32.7 kg/m^2^), and self-reported bra size of 36D (range, C-DDD) with Regnault grade 2 ptosis (range, 0-3). Twelve patients (71%) had invasive breast cancer, with 7 undergoing neoadjuvant chemotherapy and 2 requiring postmastectomy radiation therapy (PMRT).

**Table 1.** Patient Demographics

	*N*	Average	Low	High
Total patients	17			
Total breasts	31			
Bilateral reconstructions	14			
Unilateral reconstructions	3			
Patient age, years	-	50	33	68
Body mass index, kg/m^2^	-	27.4	20.4	32.7
Follow-up, months	-	5.1	0.9	13.1
Preoperative breast characteristics				
Bra cup size	-	36D	C	DDD
Regnault ptosis grade	-	2	0	3
Mastectomy weight, grams	-	683	400	1154
Existing breast scars	6			
Breast scars incorporated into bell	3			
Oncology				
Prophylactic surgery	4 (24%)			
In-situ cancer disease	1 (6%)			
Invasive cancer	12 (71%)			
Neoadjuvant chemotherapy	7 (41%)			
Postmastectomy chemotherapy	1 (6%)			
Postmastectomy radiation therapy	2 (12%)			

Of the 31 planned bell pattern breasts, 28 (90%) underwent bell pattern excision, and 3 were converted to a different skin excision pattern due to prereconstruction ischemia (1 breast—modified transverse pattern), or unplanned oncologic skin resection (2 breasts—stair step pattern) (Table 2). After breast flap vascular assessment with ICGLA but prior to bell pattern incision commitment, 9 breasts showed areas of poor flap perfusion. Of these 9 breasts, the bell pattern was able to accommodate excision of the hypoperfused areas in 8 breasts.

**Table 2.** Operative Details

	*N*	%	Low	High
Planned bell excision (breasts)	31	100		
Actual bell excision (breasts)	28	90		
Bell pattern accommodated flap ischemia	8	26		
Alternative excision due to ischemia	1	3		
Alternative excision due to cancer resection	2	6		
Planned expander (breasts)	8	26		
Planned DTI (breasts)	23	74		
Actual DTI (breasts)	21	91		
Unplanned expander (breasts)	2	9		
Unplanned TE due to ischemia	1	4		
Unplanned TEBR due to cancer resection	1	4		
	*N*	Average	Low	High
Prosthesis volume at index surgery				
Permanent silicone implant volume, cc	21	550	375	700
Expander intraoperative fill, cc air	10	375	100	500
Implant volume after exchange	8	664	520	750
OR cut time (bilateral cases only)				
DTI with bell excision, minutes	8	219	160	300
Expanders with bell excision, minutes	3	245	210	275

DTI, direct to implant; OR, operating room; TE, tissue expander; TEBR, tissue-expander breast reconstruction.

In 8 breasts, 2-stage tissue expander breast reconstruction had been planned preoperatively. Whereas 23 breasts were planned as DTI (smooth round silicone) reconstructions, only 21 implants were placed. Two planned DTI breasts were converted to TEBR intraoperatively (1 due to preconstruction ischemia and 1 due to unplanned oncologic skin resection).

**Figure 3.**
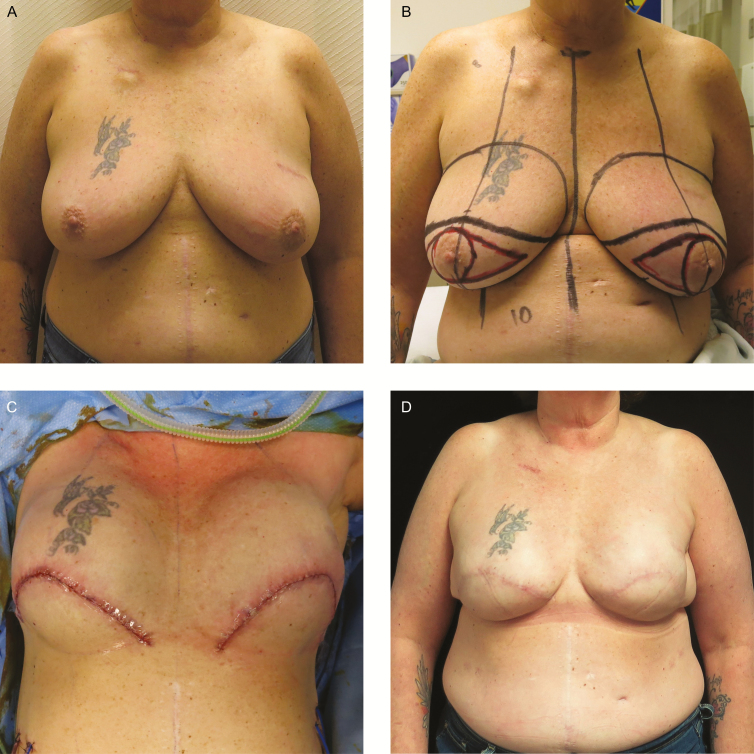
A 55-year-old woman with remote history of left lumpectomy, current obesity, and recent smoking cessation presented with left breast invasive ductal carcinoma and a single lymph node positivity and CHEK2 oncogene mutation. (A) After neoadjuvant chemotherapy. Note lumpectomy scar in upper outer quadrant. (B) Preoperative markings. (C) On-table wound closure following bilateral non-nipple-sparing mastectomy with left axillary node dissection and bell pattern skin excision with direct to permanent implant (Allergan Natrelle Inspira Cohesive Full profile 650cc silicone implants bilaterally) breast reconstruction. (D) Seven-month postoperative photograph. No major or minor complications were experienced.

Six breasts had pre-existing breast scars (including a previous inverted-T scar) of which the bell pattern was able to incorporate all or the majority of the scars in 3 breasts. Of the 3 breasts with scars outside the excision pattern, none developed flap ischemia, skin necrosis, or breast wounds (Figure 3).

**Figure 4.**
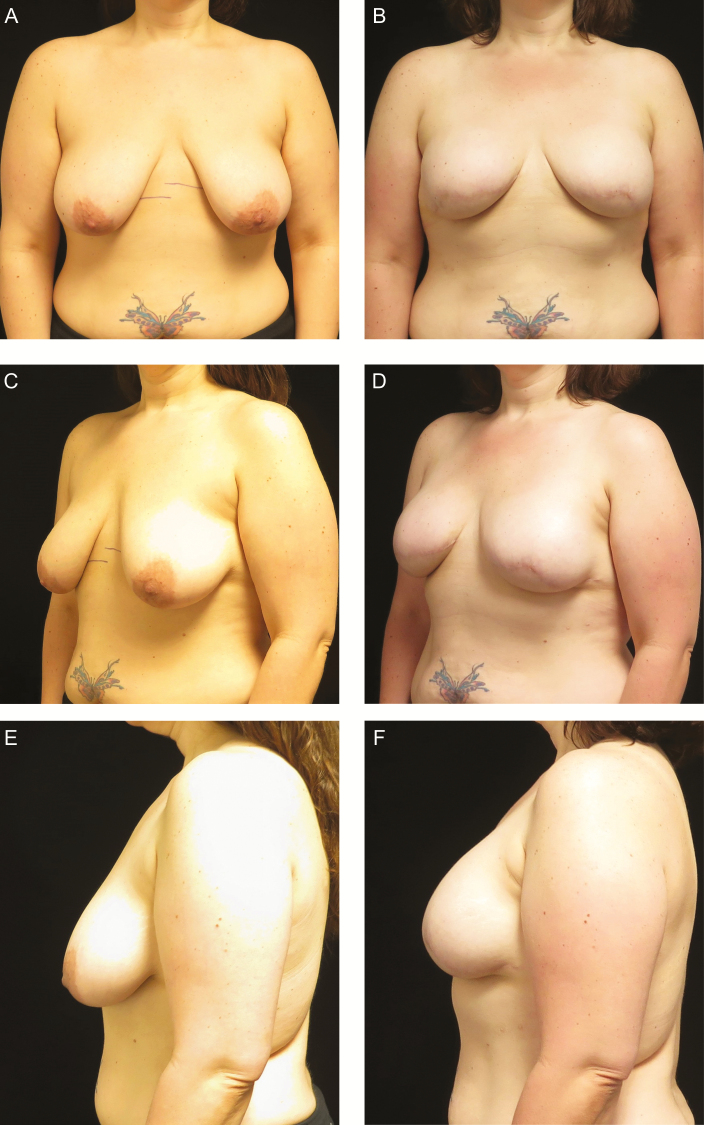
A 40-year-old woman with CHEK2 oncogene mutation desiring bilateral prophylactic mastectomies with direct-to-permanent implant reconstruction. (A, C, E) Preoperative photographs. (B, D, F) Postoperative photographs 1 year after bilateral prepectoral Allergan Natrelle Inspira Cohesive Full profile 520cc silicone implants.

**Figure 5.**
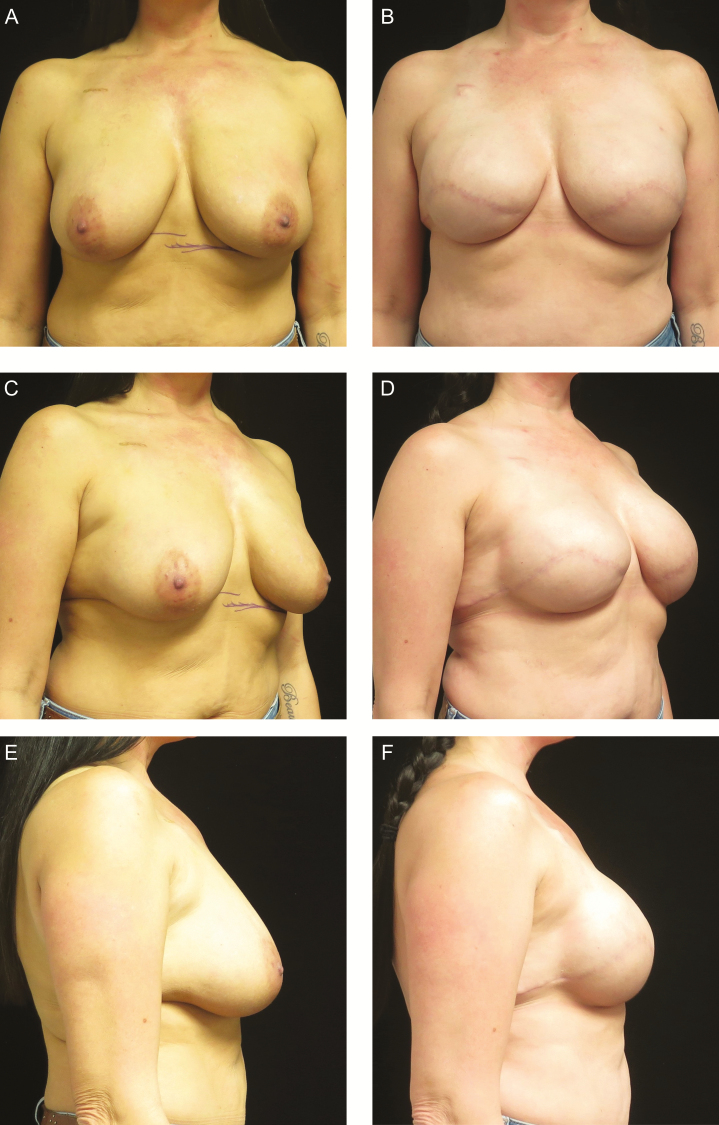
A 49-year-old woman with left breast invasive ductal carcinoma in close proximity to areola, who underwent neoadjuvant chemotherapy and desired more cleavage and superior pole fullness with her reconstruction. A 2-stage reconstruction was planned. (A, C, E) Preoperative photographs. (B, D, F) Postoperative photographs taken 7 months after index surgery and 4 months after exchange of prepectoral expanders for Allergan Natrelle Inspira Soft Touch Extra projection 750cc silicone implants.

Patients undergoing DTI reconstruction received an average implant volume of 550cc (range, 375-700cc) (Figure 4). Tissue expanders were filled with 375cc of air on average (range, 100-500cc) with subsequent second stage final implant volume of 664cc (range, 520-750cc) (Figure 5).

No major complications occurred in breasts undergoing bell pattern excision, regardless of DTI or TEBR (Table 3). Additionally, there were no unplanned operations involving the bell pattern. A single breast that required intraoperative incision conversion to a stair-step pattern due to oncologic resection (not a bell pattern) developed a late implant infection necessitating implant removal without replacement. Notably, despite the patient’s postoperative resumption of nicotine, the same patient’s contralateral bell pattern DTI breast healed without complication. No patients experienced a delay in postoperative cancer treatments.

**Table 3.** Outcomes of Bell Pattern Skin-Reducing Reconstructions (*N* = 28)

	*N*	%
Major complication	0	0
Full thickness skin necrosis	0	0
Major wound dehiscence	0	0
Hematoma (any)	0	0
Infection (any)	0	0
Unplanned return to OR	0	0
Implant exposure	0	0
Implant explantation	0	0
Delay in PM oncologic treatment	0	0
Minor complication	9	32
Seroma requiring aspiration	5	18
Minor incision wound	3	11
Superficial skin necrosis	1	4
Patient request dogear revision	0	0

OR, operating room; PM, post-mastectomy.

There were several minor complications, the most common of which was seroma necessitating aspiration occurring in 5 breasts (18%) and none required catheter placement. Curiously, 4 of these seromas were expander reconstructions. One breast (4%) developed a 2 × 1 cm area of partial skin necrosis that healed without intervention, and 3 other breasts (11%) in 2 patients developed suture knot spitting that healed with dry gauze dressing changes and no further complications (1 patient accounted for 2 breasts had active plaque psoriasis which likely contributed). No patients requested nor underwent mastectomy scar revision.

The average operative time for bilateral bell pattern IBBR (219 minutes DTI and 245 minutes TEBR) compares favorably to the author’s (W.B.A.) unpublished average “cut-time” for bilateral Wise pattern IBBR (262 minutes).

Although no validated instrument was utilized, patients reported high satisfaction at follow-up visits.

## DISCUSSION

In women with large or ptotic breasts requiring non-nipple-sparing SRM and requesting immediate IBBR, these results show the bell pattern for skin excision is a flexible and safe alternative to traditional skin resection patterns. The bell pattern allows surgeons to perform single-stage DTI reconstruction in this high-risk population with few complications.

As the most frequently published and utilized SRM pattern, the Wise pattern with or without a caudally based dermal flap^17–25^ was benchmarked for comparison. Due to variation in techniques (above/below muscle, DTI/TEBR, with or without ADM or dermal flaps), a true direct comparison of complication rates is difficult. Additionally, differences between cohorts vis-à-vis surgical risk factors likely further frustrate direct comparison between complication rates of different techniques. The rate of major complications and implant failure in our series (zero) compares favorably to rates published for Wise pattern SRM: major complications in 30.3% of DTI and 20.3% of TEBR,^6^ and implant failure in 2.25% to 14.3%.^6,18^ Inbal et al^22^ report a 50% chance of reoperation for patients who experience a major complication after Wise pattern immediate IBBR and had a 7.7% overall failure rate. For context, Jones et al^27^ reported a 2.7% failure rate for prepectoral DTI when utilizing any excision pattern. In a systematic review,^6^ pooled complication rates for IBBR utilizing Wise pattern SRM were reported for full thickness skin necrosis (9.69% DTI vs 4.69% TEBR), delayed wound healing (3% DTI vs 0.78% TEBR), and infection (2.25% DTI vs 3.91% TEBR). We report no full thickness skin loss and no infections.

With the bell pattern, the incidence of seromas requiring aspiration (18%) was higher than reported rates of seroma in immediate IBBR utilizing human ADM (12.7% for partial submuscular^28^ and 11.3% for prepectoral^27^), and in immediate IBBR with Wise pattern SRM (1.15% DTI vs 4.69% TEBR^6^). This difference is likely a reflection of the author’s preference to attempt aspiration for periprosthetic serous fluid at every expansion even if no fluid is clinically detectable. As the definition of what constitutes a seroma remains elusive, the aspiration of any serous fluid was recorded as a seroma. Typical seromas were less than 20cc aspirated during the first expansion without subsequent recurrence. Only 1 patient, accounting for 2 breasts, developed recurrent seromas. No seromas required additional drain placement.

Compromised flap perfusion is a common complaint with Wise pattern SRM and likely relates to pattern design. Mastectomy skin flap necrosis rates have been reported to range between 5% and 30% and Wise pattern resections have identified as an independent risk factor for skin flap necrosis.^29^ The residual flaps have a smaller vascular base (by excluding the entire lower pole of the breast), must be advanced a significant distance, and cut or folded into acute angles creating focal tension vectors.^6,30^ To account for compromised flap perfusion and in an effort to limit additional tension at the T point, some authors espouse use of air-filled expanders necessitating a 2-stage reconstruction, or smaller permanent implant volumes.^21,31^ In our study, average permanent implant volume was 550cc with a high of 700cc, without any implant loss or major complication. In contradistinction, De Vita et al^32^ reported an average implant volume of 478cc utilizing a Wise pattern SRM and had a significant major complication rate of 29%. Irwin et al^21^ obtained a 420cc median fill with partial submuscular Wise pattern DTI reconstruction but had an implant failure rate of 3.8%. With the bell pattern, there are no inherent corners requiring advancement, nor is the most at-risk skin located at the caudal aspect of the reconstructed breast mound where the weight of the prosthesis further compounds hypoperfusion. With the bell pattern, if wound dehiscence were to occur, the bell design would better facilitate wound debridement and flap advancement than an inverted-T scar.

ICGLA was used in all cases and has been shown to decrease mastectomy flap necrosis and reoperation rates,^7^ and likely contributes to our low complication rates. We recognize criticism regarding additional cost to every surgery, and some authors recommend limiting ICG use to high-risk patients.^5^

Often not reported in the literature but of prime practical importance, we report the frequency of intraoperative incision adjustment necessary to execute the preoperative planned reconstruction (TEBR or DTI). The majority of patients were able to proceed as planned with bell pattern SRM and DTI reconstruction even when areas of hypoperfusion were identified on ICGLA. Once committing to additional bell pattern incisions, only 1 patient developed a small area of partial skin necrosis and no patients developed full thickness skin necrosis, implying the additional bell incisions did not further compromise flap perfusion. This was also confirmed by repeat ICGLA. We hypothesis the low frequency of wounds relates to better overall flap perfusion due to a broader vascular base, lack of acute angles, dispersion of tension vectors, and avoidance of incision placement over points of maximal tension.

**Figure 6.**
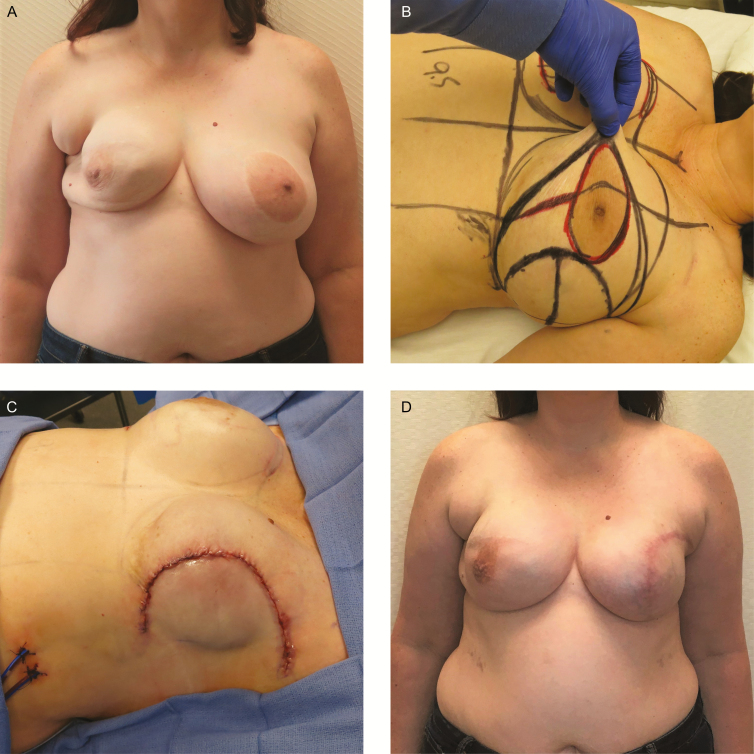
A 50-year-old woman with remote history of right breast invasive ductal carcinoma and underwent a 2-stage reconstruction with a subpectoral saline implant and left breast Wise pattern reduction for symmetry. The patient presents now with recent diagnosis of ATM oncogene mutation and desired left breast reconstruction and right breast reconstruction revision. (A) Preoperative photograph. (B) Preoperative markings for rotated bell flap. (C) Postoperative on-table result. Left breast expander filled with 400cc of air. Right breast with prepectoral conversion to Allergan Natrelle Inspira Soft-Touch full profile 695cc silicone implant with acellular dermal matrix wrap. (D) Postoperative result 6 months after index surgery and 2 months after expander exchange for permanent silicone implant.

Although the pattern did not adapt well to unplanned oncologic skin resection, it adapted well to existing breast scars. One patient presented with a previous Wise pattern breast reduction and pronounced NAC stretching (9 × 10.5 cm) (Figure 6). The vertical and inferolateral transverse limbs of the inverted T were incorporated into a “rotated” bell pattern where the base of the bell was oriented laterally forming a “C” shape on the patient’s left breast. This allowed removal of previous scar and the entire NAC, a difficult prospect with vertically oriented SRM. She developed no complications in the breast and had an appealing breast shape after expansion without a superior pole dogear.

The surgeon’s operative time with the bell pattern was lower than self-reported Wise pattern times, likely due to easier flap adjustment based on perfusion, shorter overall incision length, and minimal de-epithelialization. While not limited to SRM, Fischer et al^33^ reported average OR times for immediate IBBR of 190.7 minutes (SD 107 minutes) for DTI, and 200.4 minutes (SD 87.7 minutes) for TEBR. OR times will likely vary due to a host of variables unrelated to skin excision pattern, including but not limited to prepectoral technique, ADM use, efficiency with fluorescent angiography, and availability of surgical assistant for wound closure.

Regarding the overall aesthetic appearance of the bell pattern, no validated instruments were used to assess patient satisfaction or aesthetic outcome in our study. However, most patients reported satisfaction with the results. This is supported by the low rate of revision surgery (30%) requested by patients with prepectoral DTI reconstructions, which mirrors reported revision rates of 37% with similar reconstructions.^27^

The elliptical excision resulting in a transverse scar has been criticized for poor aesthetic outcome involving flattening of the breast apex, and residual medial and lateral dogears, particularly in larger breasted patients.^12,23^ Subjectively, the bell pattern creates a more rounded breast shape. This is thought to be due to the curvature allowing for continuously changing tension vectors rather than a single tension vector, akin to Z-plasty. Additionally, the bell pattern effectively lengthens the incisions helping to reduce redundancy. This is supported by the fact that no patients requested nor underwent mastectomy scar revision for dogears. Finally, elliptical excision limits intraoperative prosthesis fill when compared to the Wise pattern (159cc vs 197cc)^24^ making it a poor choice in women with large breasts desiring DTI reconstruction.

In breast reductions, Zhu et al^8^ showed that the Wise pattern produces a more conical overall breast shape with more projection than a modified Robertson (bell scar) technique, which may or may not translate to breast reconstruction where the implant is more likely to dictate final shape. Yet, in order to achieve this more projected breast shape with a Wise pattern, surgeons accept less implant volume, more frequent staged procedures and higher complications.^24,34^ We submit that patients may prefer a less conical breast shape with a well healed bell scar to that of a poorly healed inverted-T scar after it has healed by secondary intention. Also, the bell pattern scar is not completely foreign to the breast literature, as it closely mimics a modified Robertson breast reduction, an accepted breast scar pattern.^8,35,36^

Our study is limited by its retrospective design, small sample size, single surgeon experience, and short follow-up period. The short follow-up time makes it difficult to draw conclusions about long term complications and patient satisfaction. Our relatively smaller sample size also makes it difficult to draw conclusions about relative safety between techniques. As our goal was to ascertain safety with a novel skin excision, we excluded patients with certain known risk factors for reconstruction failure, such as active nicotine use,^37^ BMI >35,^33^ and previous history of breast radiation.^38^ However, other known risk factors were not excluded and were represented in our cohort: breast weight >600 g,^5,22,39,40^ age greater than 65,^41^ bilateral reconstructions,^42,43^ immediate DTI reconstructions,^34^ BMI >30,^33,42^ implant volume >500cc,^32^ ADM,^44^ patients requiring PMRT,^38,45^ and invasive breast disease.^46^

As for any study involving SRM, the reconstruction outcomes are inextricably linked to quality of the oncologic surgery. While all reconstructions were performed by a single plastic surgeon and thus limit generalizability, the mastectomies were performed by 8 different general surgeons at 4 different hospitals. While clearly a confounding variable, this variety may further support the efficacy and flexibility of the bell pattern.

Future areas of investigation include quantifying vascular improvement of the excision pattern relative to alternative excision patterns, expanding patient inclusion criteria to higher BMI, and adjusting the pattern to allow nipple sparing techniques.

## CONCLUSION

The bell pattern skin excision is a novel and safe technique that can and should be considered for large breasted patients desiring immediate implant-based reconstruction, including single-stage procedures. The rate of complication is low relative to current Wise pattern techniques and offers unique preoperative and intraoperative adjustability to allow for execution of the planned IBBR.
